# Drunkorexia and Emotion Regulation and Emotion Regulation Difficulties: The Mediating Effect of Disordered Eating Attitudes

**DOI:** 10.3390/ijerph18052690

**Published:** 2021-03-07

**Authors:** Vanessa Azzi, Souheil Hallit, Diana Malaeb, Sahar Obeid, Anna Brytek-Matera

**Affiliations:** 1 Faculty of Medicine and Medical Sciences, Holy Spirit University of Kaslik (USEK), Jounieh 446, Lebanon; Vanessakazzi18@outlook.com; 2 INSPECT-LB: Institut National de Sante Publique, Epidemiologie Clinique et Toxicologie-Liban, Beirut 6573-14, Lebanon; saharobeid23@hotmail.com; 3 School of Pharmacy, Lebanese International University, Beirut 1083, Lebanon; diana.malaeb@liu.edu.lb; 4 Faculty of Arts and Sciences, Holy Spirit University of Kaslik (USEK), Jounieh 446, Lebanon; 5 Institute of Psychology, University of Wroclaw, Dawida 1, 50-527 Wroclaw, Poland

**Keywords:** drunkorexia, disordered eating attitudes, emotion regulation, emotion regulation difficulties

## Abstract

Drunorexia refers to food calorie intake restriction to prevent weight gain and the desire to enhance the more extensive intoxicating effects of alcohol. The present study aimed to investigate the association of drunkorexia with emotion regulation as well as emotion regulation difficulties across the Lebanese population, and assess disordered eating attitudes as a potential mediator of these relationships. The cross-sectional study enrolled participants (*n* = 258) from all Lebanese districts. The study was performed through an online survey based on a self-designed and structured questionnaire. The Drunkorexia Motives and Behaviors Scales (DMBS), the College Life Alcohol Salience Scale (CLASS), the Difficulties in Emotion Regulation Scale (DERS-16), the Emotion Regulation Questionnaire (ERQ) and the Eating Attitudes Test (EAT-26) were used in the present study. The results showed that higher EAT-26 total scores (more disordered eating attitudes) (*B* = 0.16) and higher DERS-16 total score (*B* = 0.30) were significantly associated with more drunkorexia motives. Also, higher EAT-26 total scores (*B* = 0.09) and higher DERS-16 total score (*B* = 0.17) were significantly associated with more drunkorexia behaviors. In addition, higher EAT-26 total scores (*B* = 0.10) and higher DERS-26 total score (*B* = 0.36) were significantly associated with more drunkorexia fails. Furthermore, higher EAT-26 total scores (*B* = 0.07), and higher DERS-16 total score (*B* = 0.37) were significantly associated with more drunkorexia during an alcohol consumption event. Higher EAT-26 total scores (*B* = 0.09), and higher DERS-16 total score (*B* = 0.22) were significantly associated with more post-drinking compensation. Higher EAT-26 total scores (*B* = 0.21), higher DERS-16 total scores (*B* = 0.65) and higher emotion regulation (*B* = 0.33) were significantly associated with higher CLASS scores. The results showed that EAT-26 total scores partially mediated the association between DERS-16 total score and drunkorexia motives (25.20%), between DERS-16 total score and drunkorexia behaviors (25.16%), between DERS-16 total score and drunkorexia fails (106.87%), between DERS-16 total score and drunkorexia during an alcohol consumption event (11.84%), between DERS-16 total score and post-drinking compensation (22.55%), between ERQ total score and college life alcohol salience (8.35%) and between DERS-16 total score and college life alcohol salience (20.14%). This study highlighted that only emotional regulation difficulties were associated with drunkorexia, whereas emotional regulation was not significantly associated with such behavior.

## 1. Introduction

Over the last few years, researchers have sounded the alarm about an upsurge in binge drinking behavior. Along with this disorder, society has witnessed the emergence of a new phenomenon, a recurrent inappropriate compensatory behavior to avoid weight gain from consuming alcohol [1]. Therefore, calorie restriction to prevent weight gain and a desire to enhance the intoxicating effects of alcohol have been identified as the primary motives underlying this disorder [2]. This behavioral pattern has been called “Drunkorexia”, a non-medical term firstly introduced in 2008 by Chambers [3] and later labeled “alcoholimia”, by the medical community [4,5,6]. It shares features found in eating disorders (anorexia nervosa and bulimia nervosa)—skipping meals, fasting, self-induced vomiting, engaging in strenuous sports, using laxatives, purging—with the added benefit of drinking alcohol [7]. In fact, several studies have illustrated the high prevalence of drunkorexia among college students, mainly women [8,9,10,11]; however, due to variations in measurement tools, it has been challenging to pinpoint the exact percentage of college students who engage in these behavioral patterns [7]. For instance, one indicated that of 4271 students, 39% reported purposefully restricting food consumption before drinking. Undoubtedly, rates of drunkorexia among college women were particularly concerning: researchers found that of those who reported restricting calories due to weight concerns, 47% were women, whereas 32% were males [9]. Likewise, Bryant et al. [12] replicated these findings, showing that women were significantly more likely to consume fewer calories during at least one meal before alcohol consumption than their male counterparts, mainly to purge calories from alcoholic beverages [4,12]. Nevertheless, only one Italian study explored the prevalence of drunkorexia among the general population by including both college and non-college students. The findings revealed an upsurge of this behavior across young adults of 18 to 26 years old, along with a significant association with both alcohol abuse and cocaine use, though no gender-related differences were yet to be found [13].

While most of the conducted studies focus mainly on drunkorexia definition and its adverse outcomes such as malnutrition, nausea, syncope, physical assaults, unprotected sexual activities, depression, anxiety, and cognitive impairment [9,14,15], there is a scarcity of research explaining young adults’ propensity to partake in perilous behavioral patterns [15,16,17,18]. Emotional dysfunction has been amongst speculated psychological motives, yet it has not been thoroughly examined. For several years, the literature emphasized the role of overcoming emotional arousal and controlling emotional expression as core aspects of Emotional Regulation; however, in 2004, Gratz and Roemer [19] pioneered a novel conceptualization that outlines fundamental aspects of emotional regulation: awareness, understanding, and acceptance of emotions, the ability to control impulsive behaviors and to act following desired goals even when experiencing negative emotions, and the ability to evaluate functional emotional regulation strategies to reduce emotional arousal. Therefore, an absence of any of these cognitive steps would result in emotional dysregulation or poor control [19,20]. Furthermore, other research has established an association between emotional dysregulation, alcohol abuse, and eating disorders [21,22,23]. However, only two studies have assessed the emotional management in drunkorexia. The findings revealed that adolescents who indulge in drunkorexia behavior lacked adaptive measures to effectively control impulsive behavior when undergoing negative emotional states and experienced difficulties in understanding their emotions. Therefore, this dysfunctional behavior is presumed to be a way to cope with intense emotional states [5,6].

Other correlates of eating disorders have also been suggested: individuals undergoing emotional dysregulation may indulge in deleterious eating patterns, such as exacerbated weight control strategies to manage anger, distress, frustration, and to compensate for the lack of emotional expression [24]. Thus, in the context of previous studies, it has become compelling to thoroughly investigate whether individuals who are unable to reduce their emotional arousals effectively and express their negative emotions adaptively are more likely to engage in eating disordered behaviors and compensatory weight control strategies, a core component of drunkorexia.

Moreover, previous research has highlighted the association between eating disorder patterns and drunkorexia. For instance, an Italian study revealed that young female adults diagnosed with eating disorders were more subject to indulge in Drunkorexia behaviors [25]. Furthermore, earlier research on eating disorders suggest that individuals with several types of eating disorders are characterized by emotional regulation difficulties in addition to interoceptive deficits [26,27]. 

Motives underlying drunkorexia have not been fully examined, especially exploratory analyses of psychological, emotional predictors, and eating disorders. Specifically, a study conducted in Lebanese college students showed that 21.2% of college students were at risk of developing an eating disorder, while 11.4% had already been diagnosed with an eating disorder [28]. However, to the best of our knowledge, no other nationally conducted study has yet considered the mediating role of disordered eating attitudes in the relationship between drunkorexia and emotion regulation as well as emotion regulation difficulties. Hence, the present study aimed to investigate the association of drunkorexia with emotion regulation (measured by the Emotion Regulation Questionnaire) as well as emotion regulation difficulties (measured by the Difficulties in Emotion Regulation Scale) the across the Lebanese population, and assess disordered eating attitudes as a potential mediator of these relationships.

## 2. Materials and Methods

### 2.1. Study Design and Procedure

This was a descriptive cross-sectional observational study based on an online anonymous survey. It was conducted from September through December 2020. The voluntary survey was conducted on Lebanese population located in all Governorates of Lebanon (Beirut, Mount Lebanon, North, South, and Bekaa). To minimize interviewer risks as well as meeting lockdown restrictions enforced by the Lebanese Government, a snowball sampling method was used for the survey using online Google forms (https://docs.google.com/forms/d/19057GfbGBkdUWSm38pv9mzmBGYq9jU53WAf5CAK6M-Q/edit?usp=forms_home&ths=true, accessed on 5 February 2021). The survey was distributed via social applications including WhatsApp, LinkedIn, and Facebook. As previously documented in the literature, online questionnaires create an opportunity to collect data on a national scale and sampling multiple subgroups of individuals [29,30]. All invited participants were above 18 years of age. All scales used in the present study (except the Emotion Regulation Questionnaire and the Eating Attitudes Test) underwent a forward-back translational process.

### 2.2. Minimal Sample Size Calculation

From a previous study where 79.1% of respondents reported engaging in drunkorexia [31] and the absence of similar studies in Lebanon, the minimal sample size was calculated according to the Epi Info software version 7.2 (population survey) indicating 254 participants are needed to ensure a two-sided confidence level of 0.05 for detecting significance. 

### 2.3. Questionnaires

The self-administered questionnaire used was in Arabic, the native language of Lebanon, and required approximately 20 min to complete. Participants were asked to fill it out without assistance to avoid potential influence when answering the questions. The anonymity of the participants was guaranteed. 

The first part of the questionnaire evaluated participants sociodemographic information age; marital status, and educational level. Educational level was categorized into complementary, secondary, and university level. In addition, the household crowding index was calculated by dividing the number of persons living in the house by the number of rooms, excluding the bathroom and the kitchen [32].

The second part of the questionnaire was composed of different scales from the following:

(1) *The Drunkorexia Motives and Behaviors Scales(DMBS)*: The DMBS [33] contains a total of 52 items that evaluate participants’ engagement in drunkorexia. Each item includes the prompt “Rate the frequency of each statement” and the items are on a Likert type-scale including never (1), seldom (2), sometimes (3), often (4), and very often (5). It includes five factors: Drunkorexia Motives (15 items) classified into the reasons why individuals engage in drunkorexia (example: “Because it helps me enjoy a party”), Drunkorexia Behaviors (8 items) that relate to different behaviors associated with drunkorexia (example: “By exercising more than normal”), Drunkorexia Fails (10 items) classified into avoidance/approach behaviors used when drunkorexia fails (example: “Avoid drinking beer” and “Drink hard liquor because it has lower calories”), drunkorexia During an Alcohol Consumption Event (nine items) that related to drinking behaviors and calories (example: “Drink as much as your friends drink” and “Will make yourself throw up so that you don’t have as many calories in your system”), and Post-Drinking Compensation (10 items) classified into behavior following a night of drinking (example: “Purge or vomit to get rid of the extra calories”) [33]. In the present study, the Cronbach’s α values of the five subscales were: Drunkorexia motives α = 0.957, Drunkorexia behaviors α = 0.950, Drunkorexia fails α = 0.968, Drunkorexia during alcohol comsumption event α = 0.944 and Post-drinking compensation α = 0.952.
(2) *The College Life Alcohol Salience Scale (CLASS)*: The CLASS [34] evaluated subjects’ views in regards to how fundamental alcohol consumption is within the university culture. The CLASS consists of 15 items with the prompt “To what extent do you agree with the following statements based on alcohol use during college?” followed by statements, such as “Parties with alcohol are an integral part of college life” and “To become drunk is a college rite of passage.” Participants responded to items using a 5-point Likert scale with choices including, “Strongly Disagree”, “Disagree”, “Neither Agree nor Disagree”, “Agree”, and “Strongly Agree” [34]. In the present study, the Cronbach’s α values was 0.971.
(3) *The Difficulties in Emotion Regulation Scale (DERS-16)*: It is a 16-item scale that assesses emotion regulation difficulties [35]. Items are graded using a 5-point Likert scale. Higher scores reflect more emotion regulation difficulties. Within the scale are five subscales: clarity (lack of emotional clarity; two items), goals (difficulties engaging in goal-directed behavior; three items), impulse (impulse control difficulties; three items), non-acceptance (non-acceptance of emotional responses; three items) and strategies (limited access to effective emotion regulation strategies; five items). This scale has good reliability [36], which was confirmed in our sample (total: α = 0.961, clarity: α = 0.880; goals: α = 0.883; impulse: α = 0.895; non-acceptance: α = 0.897; strategies: α = 0.863).
(4) *Emotion Regulation Questionnaire (ERQ)***:** Validated in Arabic [37], it is composed of 10 items that measure whether a respondent uses cognitive reappraisal or expressive suppression to regulate their emotions [38]. Answers options varied between 1 (strongly disagree) and 7 (strongly agree). Higher scores reflect a larger use of the concerned emotion regulation strategy [38]. In the present study study, the Cronbach’s alpha was 0.905.
(5) *The Eating Attitudes Test (EAT-26)*: The EAT-26 [39], validated in Arabic [40], is a 26-item questionnaire that is used to measure irregular eating behaviors and concerns about weight. The EAT has three subscales: Dieting, Bulimia and Food Preoccupation, and Oral Control. Participants responded to the items using a 6-point Likert scale with choices including, “Never”, “Rarely”, “Sometimes”, “Often”, “Very Often”, and “Always.” The choices of “Never”, “Rarely”, and “Sometimes” were scored as zero and the rest of the choices were scored 1, 2, and 3 respectively. A score above 20 is viewed as a sign of an eating disorder problem. The EAT is scored for a total of all of the items (example items: “I am preoccupied with a desire to be thinner”, “I feel extremely guilty after eating”, and “I have the impulse to vomit after meals”). In the current sample, Cronbach’s alpha for the total scale was 0.89.

### 2.4. Statistical Analysis

Data analysis was performed with SPSS software version 25 (IBM, New York, NY, USA). The drunkorexia motives score, drunkorexia behaviors score, drunkorexia fails score, drunkorexia during alcohol consumption on events score, drunkorexia post drinking compensation score, and the CLASS total score taken as continuous variables and anxiety, stress, educational level, the EAT-26 total score as independent variables. Pearson correlation was used for linear correlation between continuous variables. The Student t-test and ANOVA F tests were used for categorical variables with two or more levels, respectively. The effect size of the linear regressions was calculated using this formula: $f2=\frac{R2}{1-R2}$ [41] where R^2^ is the percentage of variance of the dependent variable explained by the independent variables entered in the model. Cohen classified the effect size as small (f^2^ ≥ 0.02), medium (f^2^ ≥ 0.15) and large (f^2^ ≥ 0.35).

### 2.5. Mediation Analysis

The PROCESS SPSS Macro version 3.4, model four [42] was used to check for a possible mediating effect of disordered eating attitudes in the association between emotion regulation as well as emotion regulation difficulties (taken as independent variables) and drunkorexia (each aspect taken as a dependent variable). A significant mediation was determined if the confidence interval (CI) around the indirect effect did not include zero [42]. The covariates that were included in the mediation model were those that had an effect size or a correlation coefficient >0.24 to obtain parsimonious models. Significance was set at *p* < 0.05.

## 3. Results

### 3.1. Sociodemographic and Other Characteristics of the Participants

A total of 258 (75.88%) out of 340 participants participated in this study. The mean age of the sample was 26.96 ± 9.39 years, with 78.7% males. The means and standard deviations of the drunkorexia scores, as well as other sociodemographic characteristics of the participants, are summarized in Table 1.

### 3.2. Bivariate Analysis

Higher levels of emotion regulation, higher difficulties in emotion regulation and more disordered eating attitudes were significantly associated with more drunkorexia motives, drunkorexia behaviors, drunkorexia fails, drunkorexia during alcohol consumption on events, drunkorexia post-drinking compensation and college life alcohol salience scores (Table 2). 

None of the sociodemographic variables showed a significant association with any of the drunkorexia scores (Table 3). 

### 3.3. Multivariable Analysis

Results of the multivariable analysis are summarized in Table 4, taking each drunkorexia score as the dependent variable, after adjusting across variables that showed a correlation or an effect size > I 0.24 I in the bivariate analysis. Independent variables with a higher standardized Beta value would have a greater magintude of effect on the dependent variable. The results of the first linear regression, taking the drunkorexia motives score as the dependent variable, showed that higher EAT-26 total scores (*B* = 0.16; 95% CI 0.11–0.22; *p* < 0.001) and higher DERS-16 total score (*B* = 0.30; 95% CI 0.20–0.30; *p* < 0.001) were significantly associated with more drunkorexia motives (Table 4, Model 1).

The results of the second linear regression, taking the drunkorexia behaviors score as the dependent variable, showed that higher EAT-26 total scores (*B* = 0.09; 95% CI 0.05–0.12; *p* < 0.001) and higher DERS-16 total score (*B* = 0.17; 95% CI 0.11–0.23; *p* < 0.001) were significantly associated with more drunkorexia behaviors (Table 4, Model 2).

The results of the third linear regression, taking the drunkorexia fails score as the dependent variable, showed that higher EAT-26 total scores (*B* = 0.10; 95% CI 0.05–0.16; *p* < 0.001) and higher DERS-16 total score (*B* = 0.36; 95% CI 0.26–0.46; *p* < 0.001) were significantly associated with more drunkorexia fails (Table 4, Model 3).

The results of the fourth linear regression, taking the drunkorexia during alcohol consumption on events score as the dependent variable, showed that higher EAT-26 total scores (*B* = 0.07; 95% CI 0.03–0.11; *p* < 0.001), and higher DERS-16 total score (*B* = 0.37; 95% CI 0.29–0.45; *p* < 0.001) were significantly associated with more drunkorexia during alcohol consumption on events (Table 4, Model 4).

The results of the fifth linear regression, taking the drunkorexia post drinking compensation score as the dependent variable, showed that higher EAT-26 total scores (*B* = 0.09; 95% CI 0.03–0.14; *p* < 0.001), and higher DERS-16 total score (*B* = 0.22; 95% CI 0.12–0.31; *p* < 0.001) were significantly associated with more drunkorexia post drinking compensation (Table 4, Model 5).

The results of the sixth linear regression, taking the college life alcohol salience score as the dependent variable, showed that higher EAT-26 total scores (*B* = 0.21; 95% CI 0.11–0.30; *p* < 0.001), higher DERS-16 total scores (*B* = 0.65; 95% CI 0.45–0.86; *p* < 0.001) and higher emotional regulation (*B* = 0.33; 95% CI 0.10–0.57; *p* = 0.006) were significantly associated with higher college life alcohol salience scores (Table 4, Model 6).

### 3.4. Mediation Analysis

Results of the mediation analysis are summarized in Table 5, taking each drunkorexia score as the dependent variable, after adjusting across variables that showed a correlation or an effect size > I 0.24 I in the bivariate analysis. Results showed that disordered eating attitudes (EAT-26 total scores) partially mediated the association between emotional regulation difficulties (DERS-16 total score) and drunkorexia motives (25.20%), between emotional regulation difficulties (DERS-16 total score) and drunkorexia behaviors (25.16%), between emotional regulation difficulties (DERS-16 total score) and drunkorexia fails (106.87%), between emotional regulation difficulties (DERS-16 total score) and drunkorexia during alcohol consumption on events (11.84%), between emotional regulation difficulties (DERS-16 total score) and drunkorexia post-drinking compensation (22.55%), between emotional regulation (ERQ total score) and college life alcohol salience (8.35%) and between emotional regulation difficulties (DERS-16 total score) and college life alcohol salience (20.14%). 

## 4. Discussion

Our findings revealed that emotional regulation difficulties, assessed through DERS-16 scale, were significantly correlated with all drunkorexia aspects (drunkorexia motives, drunkorexia behaviors, drunkorexia fails, drunkorexia during an alcohol consumption event and post-drinking compensation) and college life alcohol salience. For instance, emotional distress, such as depressive and anxious symptoms, were associated with drunkorexia behaviors. These findings were considered in light of several motives triggering these maladaptive patterns: the fear of weight gain due to calories in alcoholic beverages [14,43], distraction, and discomfort from aversive emotional states associated with self-criticism of shape [22,44,45,46]. Indeed, indulging in self-imposed inappropriate restrictive patterns may allow them to gain a sense of control over a threatening situation (increase in weight), thus, reducing their inner anxiety and find relief from this negative emotional state [1,47]. 

Prior research has also suggested that individuals who partake in disordered eating behaviors experience difficulties recognizing their inner emotional states, thus lacking differentiation between specific visceral sensations related to hunger, satiety, and their inner emotions [48,49]. For instance, previous literature highlighted that difficulties controlling impulsive behaviors and lack of emotional awareness were associated with Drunkorexia behaviors in males in response to intense negative emotions or emotional ambiguity [22]. Furthermore, in 2015, Ward and Galante [33] revealed that individuals experiencing negative emotional states are more likely to engage in problematic drinking behaviors, whether to enhance positive affect, comply with social expectations, seek peer acceptance, or reduce their inner negative emotions. Similarly, numerous theorists have postulated that lack of emotional skills may trigger young adults to engage in dysfunctional behaviour to change an intense emotional experience [23] and use disordered eating to cope with negative emotions, mainly through the avoidance and inhibition of such emotional states [50].

Second, our results showed that failing to regulate one’s emotions assessed through the ERQ scale, was only correlated with college life alcohol salience. These findings are likely in light of previous research, stating that difficulties controlling impulsive behaviors and lack of emotional awareness were significantly associated with heavy drinking patterns [22,51]. 

Interestingly, despite literature revealing that individuals who reported failing to regulate their emotions were more likely to partake in drunkorexia behaviors, our results showed no significant association between failing to regulate one’s emotions and drunkorexia aspects. Such findings are novel since, to our best knowledge, no other study has reported these results. Therefore, we hypothesize that the current findings highlight a novel concept, which has so far been overlooked or rarely stated: “Metacognition” exhibits a perseverative thinking processes that involves psychological skills, knowledge, experience, and is classified into positive and negative beliefs that dictate individual cognitions [52]. Specifically, both of these convictions lead to evaluative and speculative frustration, avoid a potential threat, and monitor particular thoughts, sometimes leading to maladaptive coping strategies if dysfunctional thought processes occur. As per positive metacognitions, they consider the usefulness of rumination and cognitive strategies (e.g., alcohol helps me gain control over my thoughts and emotions), whereas negative metacognitive beliefs concerning the conviction that one’s thoughts, executive functions, and emotions may be uncontrollable or harmful (e.g., I cannot control my drinking thoughts nor stop drinking once I start) [53]. Indeed, previous literature has underscored the fundamental motivational role of positive metacognitive beliefs in using alcohol as a coping strategy with one’s emotions and cognitions [20], similarly, to help control one’s thoughts, reduce frustration, which contribute to cognitive-emotional regulation [54]. Moreover, in 2013, Spada et al. [55] speculated that emotional dysregulation reflected the presence of underlying dysfunctional metacognition leading to dysfunctional metacognitions about alcohol consumption, thus, increased addiction. Furthermore, relating to our findings, Dragan [20] interestingly found no direct association between emotional dysregulation and alcohol consumption. However, the relationship between these two variables was only established once metacognitions, particularly positive beliefs, were considered potential mediators. Additionally, an innovative Italian study highlighted the direct association between metacognition and drunkorexia [56]. Therefore, in line with what has been previously stated, we hypothesize that dysfunctional metacognitions mediate and predict the association between Emotional regulation and drunkorexia. Since developed metacognitive abilities enhance the individual’s ability to regulate and manage his emotion, emotion dysregulation and metacognition are strongly connected [57]. Hence, we speculate that only if dysfunction in the metacognitive processes were to happen, would dysfunctional emotional regulation result in a greater risk for drunkorexia. However, additional research is warranted to validate this hypothesis.

In addition, concerning the hypothesized interactional effects, disordered eating attitudes only partially mediated the association between emotional regulation difficulties and drunkorexia. Indeed, research has emphasized that individuals diagnosed with an eating disorder are four times more likely to develop an alcohol use disorder [58]. Nevertheless, to improve understanding of this partial relation, it may be essential to explore the motives that trigger the development of such dysfunctional behaviour. Therefore, to help delineate the underlying motivations, it is important to look at the differences between drunkorexia and clinical eating disorders [59]. For instance, despite both drunkorexia and traditional eating disorders primarily focusing on restricting calories to prevent weight gain, engaging in drunkorexia behavior is strongly motivated by enhancing intoxicating alcohol effects [2], which is not a key component in traditional eating disorders. As such, it may be plausible that drunkorexia is more strongly associated with problematic alcohol consumption than eating disorders [31].

Regarding gender, our results showed no significant association with any of the drunkorexia scores, in contrast to many previous literature studies that have reported a higher prevalence of such behaviour among females [5,6,7,8]. Similarly, Hunt and Forbush [1] revealed that both disordered eating and alcohol use were significantly associated with drunkorexia in both male and female college students. Thus, additional research is warranted to explore gender differences. 

Finally, being married compared to single was significantly associated with more drunkorexia fails. To clarify, married individuals reported using more strategies to avoid alcohol use due to their failure to restrict food caloric intake before drinking [33]. One other study revealed that males had a higher drunkorexia fails score than their female counterparts, indicating that men would still engage in alcohol consumption despite failure to compensate for calories [33]. However, there is a scarcity of research exploring the association of socio-demographic characteristics with drunkorexia fails score; therefore, this field should be more thoroughly explored. 

### 4.1. Clinical Implications

By having a greater understanding of the motives triggering this harmful behavior, health care providers and psychologists would better identify and address such patterns. This study is of particular relevance for clinicians and researchers to implement effective prevention programs, understanding mechanisms that involve emotional regulation difficulties, and train individuals to develop better coping strategies for emotional and cognitive regulation.

### 4.2. Limitations

Despite the contribution of our study to the literature regarding drunkorexia, it is important to consider some of the limitations. First, all data was obtained using self-report instruments, thus, it might be possible that some participants have misreported some of the questions of the survey and thus there is a potential role of bias in the responses. Second, the study was a cross-sectional design, which may limit exploring the causal relations between the variables; thus, future studies could implement a longitudinal design to better understand the temporal nature of the study variables to increase our knowledge about the role of emotional regulation and drunkorexia. Third, we used a single item to assess drunkorexia consistent with previous studies [4,9].

## 5. Conclusions

This study’s results highlighted that only emotional regulation difficulties were associated with drunkorexia, whereas failing to regulate one’s emotions was not significantly associated with such behavior. Additionally, drunkorexia patterns were only partially mediated by disordered eating attitudes. Furthermore, in support of these findings, we speculated that dysfunctional metacognitions mediate and predict the association between emotion regulation difficulties and drunkorexia. However, in light of these results, future studies are warranted to evaluate these hypotheses. 

## Figures and Tables

**Table 1:** Sociodemographic and other characteristics of the participants (*n* = 258).

Variable	*n* (%)
Gender	
Male	203 (78.7%)
Female	55 (21.3%)
Marital status	
Single	203 (78.7%)
Married	55 (21.3%)
Education level	
Complementary or less	32 (12.4%)
Secondary	34 (13.2%)
University	192 (74.4%)
	Mean ± SD
Age (in years)	26.96 ± 9.39
Household crowding index	0.97 ± 0.51
Emotion regulation difficulties (DERS-16 total score)	27.17 ± 16.48
Emotion regulation (ERQ total score)	48.55 ± 13.33
Disordered eating attitudes (EAT-26 total score)	31.54 ± 27.78
Drunkorexia motives (DMBS)	20.88 ± 14.46
Drunkorexia behaviors (DMBS)	13.28 ± 8.64
Drunkorexia fails (DMBS)	21.50 ± 12.04
Drunkorexia during an alcohol consumption event (DMBS)	19.35 ± 10.20
Post-drinking compensation (DMBS)	19.74 ± 10.12
College life alcohol salience (CLASS total score)	62.85 ± 23.20

DERS-16 = Difficulties in Emotion Regulation Scale; ERQ = Emotion Regulation Questionnaire; EAT-26 = Eating Attitudes Test; DMBS = Drunkorexia Motives and Behaviors Scales; CLASS = College Life Alcohol Salience Scale; SD = Standard Deviation.

**Table 2:** Bivariate analysis of continuous variables associated with the drunkorexia scores.

Variable	Drunkorexia Motives	Drunkorexia Behaviors	Drunkorexia Fails	Drunkorexia during an Alcohol Consumption Event	Post-Drinking Compensation	College Life Alcohol Salience
Emotion regulation (ERQ total score)	0.267 ^a^	0.234 ^a^	0.331 ^a^	0.330 ^a^	0.158	0.475 ^a^
DERS total score	0.463 ^a^	0.405 ^a^	0.567 ^a^	0.671 ^a^	0.436 ^a^	0.656 ^a^
Lack of emotional clarity (DERS)	0.434 ^a^	0.406 ^a^	0.458 ^a^	0.588 ^a^	0.388 ^a^	0.603 ^a^
Difficulties engaging in goal-directed behavior (DERS)	0.354 ^a^	0.287 ^a^	0.439 ^a^	0.530 ^a^	0.317 ^a^	0.552 ^a^
Impulse control difficulties (DERS)	0.405 ^a^	0.306 ^a^	0.529 ^a^	0.642 ^a^	0.370 ^a^	0.625 ^a^
Limited access to effective emotion regulation strategies (DERS)	0.455 ^a^	0.400 ^a^	0.585 ^a^	0.684 ^a^	0.460 ^a^	0.640 ^a^
Non-acceptance of emotional responses (DERS)	0.461 ^a^	0.451 ^a^	0.584 ^a^	0.650 ^a^	0.463 ^a^	0.628 ^a^
Disordered eating attitudes (EAT-26 total score)	0.423 ^a^	0.369 ^a^	0.407 ^a^	0.400 ^a^	0.368 ^a^	0.439 ^a^
Age	−0.055	0.012	0.116	0.039	−0.012	−0.014
Physical activity	0.149 ^b^	0.153 ^b^	−0.005	0.008	−0.035	−0.114
Household crowding index	0.038	0.009	0.077	0.049	0.123	0.074

^a^*p* < 0.001; ^b^ *p* < 0.05; numbers indicate Pearson correlation coefficients obtained from the Pearson test. ERQ = Emotion Regulation Questionnaire; DERS-16 = Difficulties in Emotion Regulation Scale; EAT-26 = Eating Attitudes Test.

**Table 3:** Bivariate analysis of continuous variables associated with the drunkorexia scores.

Variable	Drunkorexia Motives	Drunkorexia Behaviors	Drunkorexia Fails	Drunkorexia during an Alcohol Consumption Event	Post-Drinking Compensation	College Life Alcohol Salience
Gender						
Male	21.74 ± 14.04	13.36 ± 8.52	21.97 ± 11.32	20.04 ± 10.28	19.48 ± 10.34	64.25 ± 21.51
Female	19.95 ± 14.90	13.18 ± 8.80	21.03 ± 12.78	18.66 ± 10.15	19.98 ± 9.96	61.45 ± 24.85
*p*	0.322	0.867	0.650	0.432	0.774	0.485
Effect size	0.123	0.020	0.077	0.135	0.049	0.120
Marital status						
Single	20.93 ± 14.77	13.21 ± 8.76	20.72 ± 12.01	19.67 ± 10.63	19.86 ± 10.41	63.20 ± 22.95
Married	20.67 ± 13.36	13.51 ± 8.23	24.38 ± 11.90	18.17 ± 8.53	19.27 ± 9.09	61.55 ± 24.44
*p*	0.905	0.824	0.147	0.485	0.784	0.735
Effect size	0.018	0.028	0.306	0.155	0.060	0.069
Education level						
Complementary or less	19.15 ± 14.73	11.56 ± 8.52	22.55 ± 14.16	19.55 ± 11.32	19.11 ± 11.11	60.72 ± 28.61
Secondary	25.73 ± 15.99	15.09 ± 8.71	22.52 ± 13.59	20.14 ± 9.13	20.71 ± 11.08	59.24 ± 22.79
University	20.30 ± 14.04	13.24 ± 8.63	21.08 ± 11.37	19.14 ± 10.30	19.64 ± 9.81	64.03 ± 22.32
*p*	0.101	0.253	0.818	0.918	0.874	0.637
Effect size	0.499	0.303	0.182	0.114	0.138	0.128

Student t test was used to compare between the different scores and dichotomous variables (gender and marital status), whereas ANOVA test was used to compare three or more means (between different scores and education level). None of the associations showed significance (at *p* < 0.05).

**Table 4:** Multivariable analyses.

Model 1: Drunkorexia Motives Score as the Dependent Variable				
Variable	UB	SB	*p*	95% CI
Emotion regulation (ERQ)	0.08	0.08	0.178	−0.04–0.19
Emotion regulation difficulties (DERS-16)	0.30	0.35	**<0.001**	0.20–0.40
Disordered eating attitudes (EAT-26)	0.16	0.31	**<0.001**	0.11–0.22
Variables entered in the model: ERQ total score, DERS-16 total score, EAT-26 total score, education level. R^2^ = 33%; effect size f^2^ = 0.122				
Model 2: Drunkorexia behaviors score as the dependent variable				
Variable	UB	SB	*p*	95% CI
Disordered eating attitudes (EAT-26)	0.09	0.28	**<0.001**	0.05–0.12
Emotion regulation difficulties (DERS-16)	0.17	0.33	**<0.001**	0.11–0.23
Variables entered in the model: DERS-16 total score, EAT-26 total score, education level. R^2^ = 23.4%; effect size f^2^ = 0.057				
Model 3: Drunkorexia fails score as the dependent variable				
Variable	UB	SB	*p*	95% CI
Emotion regulation difficulties (DERS-16)	0.36	0.50	**<0.001**	0.26–0.46
Disordered eating attitudes (EAT-26)	0.10	0.26	**<0.001**	0.05–0.16
Variables entered in the model: ERQ total score, DERS-16 total score, EAT-26 total score, marital status. R^2^ = 40.8%; effect size f^2^ = 0.199				
Model 4: Drunkorexia during an alcohol consumption event score as the dependent variable				
Variable	UB	SB	*p*	95% CI
Emotion regulation difficulties (DERS-16)	0.37	0.61	**<0.001**	0.29–0.45
Disordered eating attitudes (EAT-26)	0.07	0.20	**0.002**	0.03–0.11
Variables entered in the model: ERQ total score, DERS-16 total score, EAT-26 total score. R^2^ = 48.8%; effect size f^2^ = 0.312				
Model 5: Post-drinking compensation score as the dependent variable				
Variable	UB	SB	*p*	95% CI
Emotion regulation difficulties (DERS-16)	0.22	0.35	**<0.0011**	0.12–0.31
Disordered eating attitudes (EAT-26)	0.09	0.25	**0.002**	0.03–0.14
Variables entered in the model: DERS-16 total score, EAT-26 total score. R^2^ = 24.8%; effect size f^2^ = 0.065				
Model 6: College life alcohol salience score as the dependent variable				
Variable	UB	SB	*p*	95% CI
Emotion regulation difficulties (DERS-16)	0.65	0.47	**<0.0011**	0.45–0.86
Disordered eating attitudes (EAT-26)	0.21	0.26	**<0.001**	0.11–0.30
Emotion regulation (ERQ)	0.33	0.20	**0.006**	0.10–0.57
Variables entered in the model: ERQ total score, DERS-16 total score, EAT-26 total score. R^2^ = 51.7%; effect size f^2^ = 0.364				

Numbers in bold indicate significant *p*-values; UB = Unstandardized Beta; SB = Standardized Beta; CI = Confidence Interval.

**Table 5:** Mediation analysis.

Model 1: Drunkorexia Motives										
	Effect of Emotional Regulation on Disordered Eating Attitudes			Effect of Emotional Regulation and Disordered Eating Attitudes on Drunkorexia Motives			Effect of Emotional Regulation on Drunkorexia Motives			Mediating Effect of Disordered Eating Attitudes
	Beta	t	*p*	Beta	T	*p*	Beta	t	*p*	
ERQ total score	0.04 [−0.21–0.29]	0.32	0.748	0.07 [−0.05–0.18]	1.16	0.246	0.07 [−0.05–0.19]	1.20	0.230	-
EAT-26 total score				0.16 [0.11–0.22]	5.82	**<0.001**				
	R^2^ = 8.06%; effect size f^2^ = 0.006			R^2^ = 31.1%; effect size f^2^ = 0.107			R^2^ = 21.9%; effect size f^2^ = 0.05			
	Effect of emotional regulation difficulties on disordered eating attitudes			Effect of emotional regulation difficulties and disordered eating attitudes on Drunkorexia motives			Effect of emotional regulation difficulties on drunkorexia motives			
	Beta	t	*p*	Beta	T	*p*	Beta	t	*p*	
DERS-16 total score	0.45 [0.23–0.67]	4.07	**<0.001**	0.29 [0.19–0.40]	5.71	**<0.001**	0.37 [0.26–0.47]	6.94	**<0.001**	25.20%
EAT-26 total score				0.16 [0.11–0.22]	5.82	**<0.001**				
	R^2^ = 8.06%; effect size f^2^ = 0.006			R^2^ = 31.1%; effect size f^2^ = 0.107			R^2^ = 21.9%; effect size f^2^ = 0.05			
Model 2: Drunkorexia behaviors										
	Effect of emotional regulation on disordered eating attitudes			Effect of emotional regulation and disordered eating attitudes on Drunkorexia behaviors			Effect of emotional regulation on drunkorexia behaviors			
	Beta	t	*p*	Beta	T	*p*	Beta	t	*p*	
ERQ total score	0.04 [−0.21–0.29]	0.32	0.748	0.04 [−0.04–0.11]	0.98	0.328	0.04 [−0.04–0.11]	1.03	0.303	-
EAT-26 total score				0.09 [0.05–0.12]	4.82	**<0.001**				
	R^2^ = 8.06%; effect size f^2^ = 0.006			R^2^ = 23.7%; effect size f^2^ = 0.06			R^2^ = 16.7%; effect size f^2^ = 0.03			
	Effect of emotional regulation difficulties on disordered eating attitudes			Effect of emotional regulation difficulties and disordered eating attitudes on Drunkorexia behaviors			Effect of emotional regulation difficulties on Drunkorexia behaviors			
	Beta	t	*p*	Beta	T	*p*	Beta	t	*p*	
DERS-16 total score	0.45 [0.23–0.67]	4.07	**<0.001**	0.15 [0.09–0.22]	4.74	**<0.001**	0.19 [0.13–0.26]	5.87	**<0.001**	25.16%
EAT-26 total score				0.09 [0.05–0.12]	4.82	**<0.001**				
	R^2^ = 8.06%; effect size f^2^ = 0.006			R^2^ = 23.7%; effect size f^2^ = 0.06			R^2^ = 16.7%; effect size f^2^ = 0.03			
Model 3: Drunkorexia fails										
	Effect of emotional regulation on disordered eating attitudes			Effect of emotional regulation and disordered eating attitudes on Drunkorexia fails			Effect of emotional regulation on drunkorexia fails			
	Beta	t	*p*	Beta	T	*p*	Beta	t	*p*	
ERQ total score	−0.14 [−0.55–0.27]	−0.66	0.511	0.06 [−0.08–0.20]	0.82	0.414	0.04 [−0.10–0.19]	0.60	0.552	-
EAT-26 total score				0.10 [0.04–0.16]	3.49	**<0.001**				
	R^2^ = 10.7%; effect size f^2^ = 0.01			R^2^ = 38.0%; effect size f^2^ = 0.169			R^2^ = 32.3%; effect size f^2^ = 0.116			
	Effect of emotional regulation difficulties on disordered eating attitudes			Effect of emotional regulation difficulties and disordered eating attitudes on Drunkorexia fails			Effect of emotional regulation difficulties on Drunkorexia fails			
	Beta	t	*p*	Beta	T	*p*	Beta	t	*p*	
DERS-16 total score	0.64 [0.30–0.98]	3.71	**<0.001**	0.33 [0.21–0.45]	5.34	**<0.001**	0.39 [0.27–0.51]	6.47	**<0.001**	106.87%
EAT-26 total score				0.10 [0.04–0.16]	3.49	**<0.001**				
	R^2^ = 10.7%; effect size f^2^ = 0.01			R^2^ = 38.0%; effect size f^2^ = 0.169			R^2^ = 32.3%; effect size f^2^ = 0.116			
Model 4: Drunkorexia during an alcohol consumption event										
	Effect of emotional regulation on disordered eating attitudes			Effect of emotional regulation and disordered eating attitudes on Drunkorexia during an alcohol consumption event			Effect of emotional regulation on drunkorexia during an alcohol consumption event			
	Beta	t	*p*	Beta	T	*p*	Beta	t	*p*	
ERQ total score	−0.14 [−0.54–0.27]	−0.66	0.511	−0.01 [−0.12–0.09]	−0.20	0.841	−0.02 [−0.13–0.09]	−0.36	0.715	-
EAT-26 total score				0.07 [0.03–0.11]	3.09	**0.002**				
	R^2^ = 10.7%; effect size f^2^ = 0.01			R^2^ = 48.8%; effect size f^2^ = 0.312			R^2^ = 45.1%; effect size f^2^ = 0.255			
	Effect of emotional regulation difficulties on disordered eating attitudes			Effect of emotional regulation difficulties and disordered eating attitudes on Drunkorexia during an alcohol consumption event			Effect of emotional regulation difficulties on Drunkorexia during an alcohol consumption event			
	Beta	t	*p*	Beta	T	*p*	Beta	t	*p*	
DERS-16 total score	0.64 [0.30–0.98]	3.71	**<0.001**	0.38 [0.28–0.47]	8.00	**<0.001**	0.42 [0.33–0.51]	9.11	**<0.001**	11.84%
EAT-26 total score				0.07 [0.03–0.11]	3.09	**0.002**				
	R^2^ = 10.7%; effect size f^2^ = 0.01			R^2^ = 48.8%; effect size f^2^ = 0.312			R^2^ = 45.1%; effect size f^2^ = 0.255			
Model 5: Post-drinking compensation										
	Effect of emotional regulation on disordered eating attitudes			Effect of emotional regulation and disordered eating attitudes on post-drinking compensation			Effect of emotional regulation on post-drinking compensation			
	Beta	t	*p*	Beta	T	*p*	Beta	t	*p*	
ERQ total score	−0.14 [−0.55–0.27]	−0.66	0.511	−0.06 [−0.18–0.07]	−0.88	0.378	−0.07 [−0.20–0.06]	−1.03	0.304	-
EAT-26 total score				0.08 [0.03–0.14]	3.14	**0.002**				
	R^2^ = 10.7%; effect size f^2^ = 0.01			R^2^ = 25.2%; effect size f^2^ = 0.068			R^2^ = 19.6%; effect size f^2^ = 0.04			
	Effect of emotional regulation difficulties on disordered eating attitudes			Effect of emotional regulation difficulties and disordered eating attitudes on post-drinking compensation			Effect of emotional regulation difficulties on post-drinking compensation			
	Beta	t	*p*	Beta	T	*p*	Beta	t	*p*	
DERS-16 total score	0.64 [0.30–0.98]	3.71	**<0.001**	0.24 [0.13–0.35]	4.27	**<0.001**	0.30 [0.19–0.40]	5.32	**<0.001**	22.55%
EAT-26 total score				0.08 [0.03–0.14]	3.14	**0.002**				
	R^2^ = 10.7%; effect size f^2^ = 0.01			R^2^ = 25.2%; effect size f^2^ = 0.068			R^2^ = 19.6%; effect size f^2^ = 0.04			
Model 6: College life alcohol salience										
	Effect of emotional regulation on disordered eating attitudes			Effect of emotional regulation and disordered eating attitudes on college life alcohol salience			Effect of emotional regulation on college life alcohol salience			
	Beta	t	*p*	Beta	T	*p*	Beta	t	*p*	
ERQ total score	−0.14 [−0.55–0.27]	−0.66	0.511	0.33 [0.10–0.57]	2.81	**0.005**	0.31 [0.06–0.55]	2.44	**0.016**	8.35%
EAT-26 total score				0.20 [0.11–0.30]	4.11	**<0.001**				
	R^2^ = 10.7%; effect size f^2^ = 0.01			R^2^ = 51.66%; effect size f^2^ = 0.364			R^2^ = 45.45%; effect size f^2^ = 0.260			
	Effect of emotional regulation difficulties on disordered eating attitudes			Effect of emotional regulation difficulties and disordered eating attitudes on college life alcohol salience			Effect of emotional regulation difficulties on college life alcohol salience			
	Beta	t	*p*	Beta	T	*p*	Beta	t	*p*	
DERS-16 total score	0.64 [0.30–0.98]	3.71	**<0.001**	0.65 [0.45–0.86]	6.26	**<0.001**	0.78 [0.58–0.99]	7.47	**<0.001**	20.14%
EAT-26 total score				0.20 [0.11–0.30]	4.11	**<0.001**				
	R^2^ = 10.7%; effect size f^2^ = 0.01			R^2^ = 25.2%; effect size f^2^ = 0.068			R^2^ = 19.6%; effect size f^2^ = 0.04			

ERQ = Emotion Regulation Questionnaire; DERS-16 = Difficulties in Emotion Regulation Scale; EAT-26 = Eating Attitudes Test. Bold values denote statistical significance at the *p* < 0.05 level.

## Data Availability

Data is available upon a valid and reasonable request from the corresponding author (S.H.).
